# A comparison of virtual touch tissue quantification and digital rectal examination for discrimination between prostate cancer and benign prostatic hyperplasia

**DOI:** 10.2478/v10019-011-0026-3

**Published:** 2011-07-20

**Authors:** Xiaozhi Zheng, Ping Ji, Hongwei Mao, Jianqun Hu

**Affiliations:** 1 Department of Ultrasound, The Fourth Affiliated Hospital of Nantong University, China; 2 Department of Ultrasonic Diagnosis, The First Affiliated Hospital with Nanjing Medical University, China

**Keywords:** prostate cancer, benign prostatic hyperplasia, virtual touch tissue quantification, digital rectal examination, shear wave velocity

## Abstract

**Background:**

Virtual touch tissue quantification (VTTQ) is a new, promising technique for detecting the stiffness of tissues. The aim of this study is to compare the performance of VTTQ and digital rectal examination (DRE) in discrimination between prostate cancer and benign prostatic hyperplasia (BPH).

**Patients and methods:**

VTTQ was performed in 209 prostate nodular lesions of 107 patients with BPH and suspected prostate cancer before the prostate histopathologic examination. The shear wave velocity (SWV) at each nodular lesion was quantified by implementing an acoustic radiation force impulse (ARFI). The performance of VTTQ and DRE in discrimination between prostate cancer and BPH was compared. The diagnostic value of VTTQ and DRE for prostate cancer was evaluated in terms of the sensitivity, specificity, positive predictive value (PPV), negative predictive value (NPV) and accuracy.

**Results:**

Prostate cancer was detected in 57 prostate nodular lesions by histopathologic examination. The SWV values (m/s) were significantly greater in prostate cancer and BPH than in normal prostate (2.37 ± 0.94, 1.98 ± 0.82 vs. 1.34 ± 0.47). The area under the receiver operating characteristic curve (AUC) for VTTQ (SWV>2.5m/s) to differentiate prostate nodules as benign hyperplasia or malignancy was 0.86, while it was 0.67 for DRE. The diagnostic sensitivity, specificity, PPV, NPV and accuracy were 71.93 %, 87.5 %, 68.33 %, 89.26 %, 83.25 %, respectively for VTTQ (SWV>2.5m/s), whereas they were 33.33 %, 81.57 %, 40.43 %, 76.54 %, 68.42 % respectively for DRE.

**Conclusions:**

VTTQ can effectively detect the stiffness of prostate nodular lesions, which has a significantly higher performance than DRE in discrimination between prostate cancer and BPH.

## Introduction

Prostate cancer is an important health concern for men. Over the past 2 decades, prostate cancer patients have become the largest cancer population among all cancer patients in the United States and European Union countries.^1^ Together with prostate specific antigen (PSA), digital rectal examination (DRE) had been recommended as the preferred method for prostate cancer screening over the past decades.^2^,^3^ But the performance of DRE in detecting prostate abnormalities varies greatly and the agreement between examiners is low.^4^ Moreover, DRE can lead to rectal discomfort, rectal bleeding and even syncope,^4^ an alternative approach for DRE is needed.^5^,^6^

Virtual touch tissue quantification (VTTQ) is a new, promising implementation of the ultrasound acoustic radiation force impulse (ARFI) imaging, which can effectively and objectively detect the tissue stiffness without any discomfort by measuring the shear wave velocity (SWV) values.^7^ Recently, VTTQ has been used to quantify the stiffness of the liver, kidneys, pancreas, and spleen.^8^–^11^ Our previous study also demonstrated that VTTQ can easily detect the age-related changes in prostate stiffness.^12^

In the present study, we investigated the feasibility of VTTQ for quantifying the stiffness of nodular lesions of prostate cancer and BPH, and compared the performance of VTTQ and DRE in discrimination between prostate cancer and BPH, in order to explore a better strategy for the prostate palpation.

## Patients and methods

The study was approved by the local human research ethics committee and free signed informed consent was obtained from all the subjects. One hundred and seven patients (mean age: 66.7 ± 12.9 years, range: 51–83 years) with BPH and suspected prostate cancer based on abnormal findings on DRE (palpable nodular lesions), transrectal ultrasound (TRUS) (detection of hypoechoic lesions) and high serum levels of PSA (>4ng/ml) were enrolled in this study. All patients underwent prostate biopsies. The control group consisted of 40 healthy volunteers (mean age: 62.8 ± 19.7 years, range: 53–88 years). The inclusion criteria were: (a) absence of any history of focal or diffuse disease at any of the examined organs, assessed by subject’s history, clinical symptoms, electrocardiogram, laboratory data, radiology, echocardiography and computer tomography; (b) good visualization of the prostate on TRUS.

DRE were performed at the department of urology by two expert urologists independently. Before rectal palpation, the bladder was voided. As described previously^13^, the subjects were standing and supported on the forearms with the knees flexed, who were asked to strain down to facilitate palpation of the upper parts of the prostate and the seminal vesicles. All findings were recorded immediately. A firm nodular consistency was the main criterion for malignancy. Prostates with a consistency close to normal were classified as benign and were not further examined.

**FIGURE 1:**
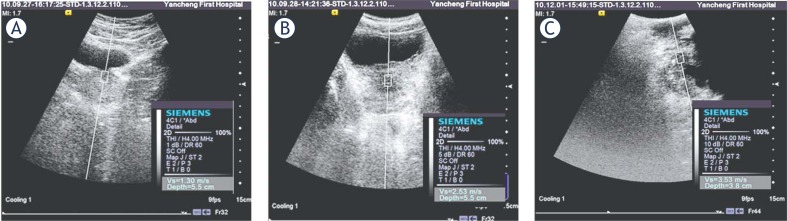
The measurement of shear wave velocity (SWV) of the normal prostate (A), benign prostatic hyperplasia (BPH) (B) and prostate cancer(C) with virtual touch tissue quantification (A and B from the abdominal view; C from the transperineal view).

Just before histopathologic examination, VTTQ were accomplished in all the patients, using a Siemens ACUSON S2000 US system (Siemens, Germany), with convex probes (4C1), tissue harmonic imaging (THI; 4MHz) and mechanical index of 1.7. Firstly, VTTQ was performed with the preliminary identification of a target region of interest (ROI) (box with fixed dimension of 1×0.5 cm) on a conventional ultrasound image. Then, an acoustic push pulse was transmitted immediately on the right side of the ROI where the SWVs were calculated and expressed with a numerical value (meter/second, m/s) as a result of multiple measurements made for the same spatial location.^7^,^9^ For the prostate study, the patients were placed in the recumbent position. The operators performed three measurements at each nodular lesion through the abdomen or perineum, after the subjects properly emptied their bladders (Figure 1).

Prostate transrectal biopsy was performed under TRUS guidance, and eight cores of tissue were collected using an 18G biopsy needle. All the specimens were fixed in 10% formaldehyde solution at a room temperature. Thereafter, they were embedded in paraffin, and cut into 5 μm-thick sections. Subsequently, the sections were stained with hematoxylin and eosin to identify prostatic carcinoma, prostatic hyperplasia, prostatitis and to assess the Gleason score of prostatic carcinoma using light microscopy (Zeiss Axiovert S 100, Jena, Germany). All results were independently evaluated by two expert pathologists.

Patient data (age, biopsy results) were collected retrospectively from the patient records. Data were expressed as the mean ± SD. Differences between the mean values of the two groups were analyzed by unpaired t tests. A McNemar test was used to compare the sensitivity, specificity, PPV, NPV and accuracy for different diagnostic criteria. A receiver operating characteristic curve (ROC) analysis was used to determine the cut off value of SWV for the diagnosis of prostate cancer, as well as to evaluate and compare the diagnostic performance of the two methods: VTTQ and DRE. Differences were considered significant at p<0.05.All statistical analysis was performed with SPSS version 13 software for Windows (SPSS Inc, Chicago, IL).

## Results

**FIGURE 2:**
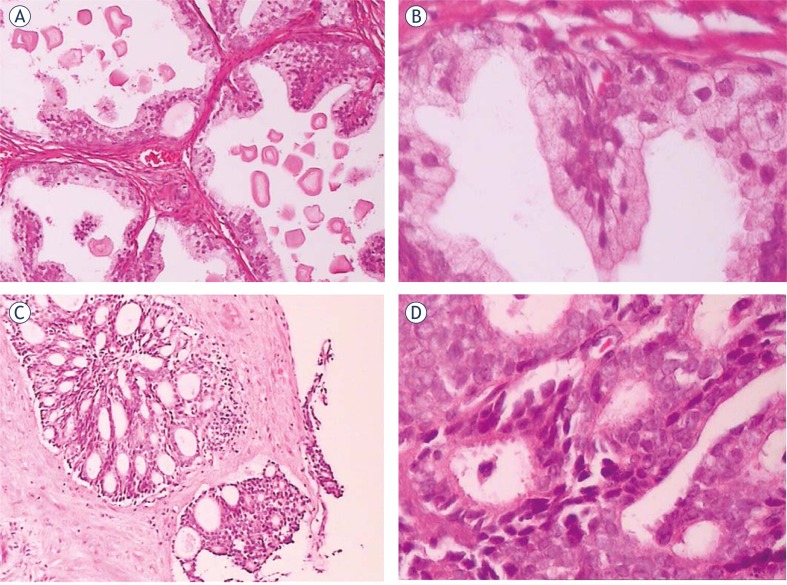
The histopathology of benign prostatic hyperplasia (BPH) (A and B), prostate cancer (C and D). (A and C: 100× magnification; B and D: 400× magnification).

Prostate cancer was detected in 57 nodular lesions of all 209 ones in 107 patients, and BPH was detected in the remaining 152 nodular lesions by the histopathologic examination. In these nodular lesions of prostate cancer, VTTQ detected 41 ones when the cutoff point of SWV was chosen at 2.5m/s, while DRE detected 19 ones per urologist in average. Acinar-type adenocarcinoma is common malignancy of the prostate, comprising more than 99% of the malignant lesions with a Gleason score of 6–10 (Figure 2). Other primary malignant prostate lesions are exceedingly rare and include germ cell tumors, malignant peripheral nerve sheath tumors and nephroblastoma. The nodular lesions of prostate cancer were mostly found at peripheral zone, while the ones of BPH were mostly found at transitional zone.

**FIGURE 3:**
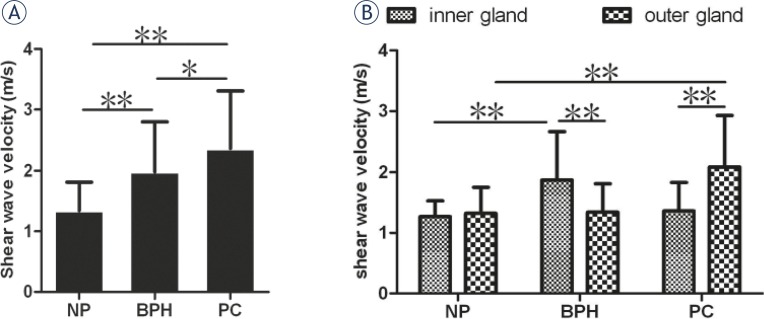
The comparison of shear wave velocity (SWV) values among benign prostatic hyperplasia (BPH), prostate cancer (PC) and the normal prostate(NP) (A) and the comparison between inner gland and outer gland (B) (*p<0.05, **p<0.01).

As shown in Figure 3, The SWV values (m/s) were significantly greater in prostate cancer and BPH than in normal prostate (2.37 ± 0.94, 1.98 ± 0.82 vs.1.34 ± 0.47). Furthermore, the SWV values were slightly greater in prostate cancer than in BPH (p<0.05). In addition, we found that the SWV values of inner gland in the patients with BPH were significantly greater than that of outer gland, while in the patients with prostate cancer, the ones of inner gland were significantly lower than that of outer gland.

**FIGURE 4:**
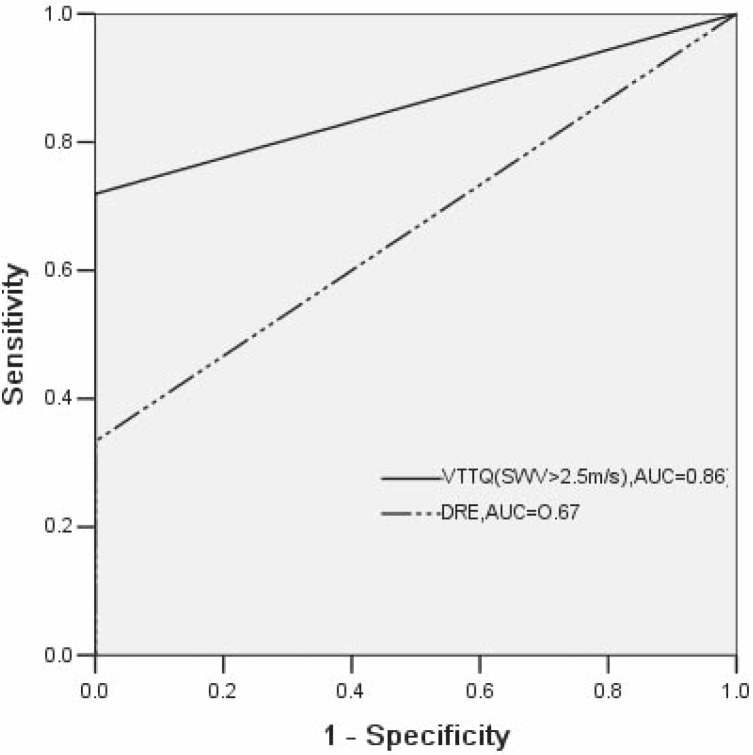
Receiver operating characteristic curve showing the performance of VTTQ (SWV >2.5m/s) and DRE in discrimination between prostate cancer and benign prostatic hyperplasia (BPH). AUC, the area under the receiver operating characteristic curve.

In order to quantify the performance of VTTQ in discrimination between prostate cancer and BPH, several cutoff values of SWV are chosen according to the area under the ROC (AUC) which is positively correlated with the discrimination performance. The definite one is 2.5 m/s at last. In the same way, the performance of VTTQ and DRE in discrimination between prostate cancer and BPH was compared. The AUC for VTTQ (SWV>2.5m/s) was 0.86, while it was 0.67 for DRE (Figure 4).

As shown in Table 1, the diagnostic sensitivity, specificity, PPV, NPV and accuracy were 71.93 %, 87.5 %, 68.33 %, 89.26 %, 83.25 %, respectively for VTTQ (SWV>2.5m/s), whereas they were 33.33 %, 81.57 %, 40.43 %,76.54 %,68.42 %, respectively for DRE. Although there were no significant differences in the specificities between VTTQ (SWV >2.5m/s) and DRE, the sensitivities, PPVs, NPVs and accuracies of VTTQ (SWV>2.5m/s) were still significantly higher than those of DRE.

## Discussion

The results presented here indicate that VTTQ can effectively detect the stiffness of prostate nodular lesions, which has a significantly higher performance in discrimination between prostate cancer and BPH than the conventionally used palpation, DRE

DRE is the most commonly used palpation technique for prostate abnormalities by detecting the changes of prostate stiffness. Although it had been recommended as one of the basic methods for prostate cancer screening, its sensitivity is not desirable. Being similar to the previous reports^14^,^15^, the diagnostic sensitivity is only 33.33% for DRE in our study. The cancer detection rate for DRE in the anterior prostate is lower than that in the peripheral region, and the difference between examiners can partly account for the low sensitivity.

ARFI imaging is a new ultrasound imaging modality to evaluate the stiffness of deep tissues by short-duration acoustic radiation forces that produce localized displacements in a “pushed” ROI^7^,^16^,^17^, and SWV is the speed of a transverse wave propagating perpendicular to the direction of tissue displacement, which is an indicative factor of tissue rigidity.^9^ Prostate is a linear, isotropic, elastic body. The stiffer the prostate, the faster the shear wave will be propagated. Our study shows that prostate cancer and BPH nodular lesions all have greater SWV values than that of the normal prostate tissue, *i.e.,* the nodular lesions of prostate cancer and BPH are stiffer than the normal prostate tissue because of their different pathological structures. This finding coincides with the results of the previous studies.^15^,^18^ In addition, our results show that the outer gland in the patients with prostate cancer has greater SWV values than that of the inner gland, and the inner gland in the patients with BPH has greater SWV values than that of the outer gland. It is known that prostate cancer often occurs in the outer gland, while BPH often occurs in the inner gland. The different originated site just explains the phenomenon of the different distribution of SWV values.

Due to the non-invasive and easily accessible nature of VTTQ, this technology makes it possible to conduct a thoroughly evaluation of prostate rigidity at an optional site. In this study, we can easily detect the stiffness by SWV measurement at any prostate nodular lesions via the abdomen or perineum; no matter they locate in inner gland and outer gland. Moreover, our previous study had demonstrated that VTTQ has a good repeatability.^12^ Therefore, the sensitivities, PPV and accuracies of VTTQ are far higher than those of DRE, although VTTQ is no significant superiority in the specificities to DRE. The AUC under the ROC further indicates that VTTQ (SWV >2.5m/s) has a significantly higher performance in the detection of prostate cancer than DRE.

Although VTTQ can be potentially an important quantitative diagnostic tool for tissue stiffness, there are some limits in the present study. For example, the stiffness of prostate cancer with different Gleason score is not evaluated, and the specimens of prostate cancer are limited. There are also some problems with the use of VTTQ for the detection of prostate cancer. The limited detected depth (maximum 5.5 cm), the fixed box dimension (1×0.5cm) of the target ROI, may become obstacles to the extensive application of this new technology.

## Conclusions

In this study, we evaluated the usefulness of VTTQ for the detection of prostate cancer. The method shows much higher sensitivity, PPV and accuracy than that of the conventionally used examination, DRE. Although several limitations mentioned above, this method still holds a considerable clinical promise, for example, a combination of VTTQ and PSA for the detection of prostate cancer.

## Figures and Tables

**TABLE 1:** The sensitivity, specificity, positive predictive value, negative predictive value and accuracy of the different examinations: DRE, VTTQ(SWV>2.5m/s).

**Modalities**	**Sensitivity**	**Specificity**	**Positive predictive value**	**Negative predictive value**	**Accuracy**
DRE	19/57(33.33%)	124/152(81.57%)	19/47(40.43%)	124/162(76.54%)	143/209(68.42%)
VTTQ	41/57(71.93%)	133/152(87.5%)	41/60(68.33%)	133/149(89.26%)	174/209(83.25%)
